# Progress in the Enantioseparation of β-Blockers by Chromatographic Methods

**DOI:** 10.3390/molecules26020468

**Published:** 2021-01-17

**Authors:** Yiwen Yang, Yehui Wang, Zongbi Bao, Qiwei Yang, Zhiguo Zhang, Qilong Ren

**Affiliations:** 1 Institute of Pharmaceutical Engineering, College of Chemical and Biological Engineering, Zhejiang University, Hangzhou 310027, China; a839493728@163.com (Y.W.); baozb@zju.edu.cn (Z.B.); vigaryang@zju.edu.cn (Q.Y.); zhiguo.zhang@zju.edu.cn (Z.Z.); renql@zju.edu.cn (Q.R.); 2 Institute of Zhejiang University-Quzhou, Quzhou 324000, China

**Keywords:** β-blockers, chromatography, enantioseparation, SFC, SMB, HPLC

## Abstract

β-adrenergic antagonists (β-blockers) with at least one chiral center are an exceedingly important class of drugs used mostly to treat cardiovascular diseases. At least 70 β-blockers have been investigated in history. However, only a few β-blockers, e.g., timolol, are clinically marketed as an optically pure enantiomer. Therefore, the separation of racemates of β-blockers is essential both in the laboratory and industry. Many approaches have been explored to obtain the single enantiomeric β-blocker, including high performance liquid chromatography, supercritical fluid chromatography and simulated moving bed chromatography. In this article, a review is presented on different chromatographic methods applied for the enantioseparation of β-blockers, covering high performance liquid chromatography (HPLC), supercritical fluid chromatography (SFC) and simulated moving bed chromatography (SMB).

## 1. Introduction

β-adrenergic antagonists, usually termed as β-blockers, are competitive antagonists of the sympathetic effects of catecholamines on beta-adrenergic receptors [1,2] with an amino-, hydroxyalkyl- side chain and at least one chiral center. Chemical structures of typical β-blockers are presented in Table 1.

β-blockers are an exceedingly important class of cardiovascular medication used mostly to treat arterial hypertension, chronic heart failure, and coronary artery disease, representing the cornerstone therapy in these cases for a long time [3]. In fact, four people at three times were awarded Noble prize in the adrenergic receptor research area, i.e., Sir Henry H. Dale being awarded Nobel prize for medicine in 1936, Sir James Black being awarded Nobel prize for medicine in 1988, Profs. Brian Kobilka and Robert Lefkowitz being awarded Nobel prize for Chemistry in 2012 [2]. Among chiral drugs, β-adrenergic blocking agents are one of the most investigated pharmaceuticals for their stereochemical impact on pharmacodynamics and pharmacokinetics [4]. Although β-blockers have been excluded from guidelines as the first-line therapy in essential hypertension, they remain the first choice in patients with heart failure, coronary artery disease, and atrial fibrillation [3]. At least 70 β-blockers have been investigated in history, as presented in Table 2, according to the literatures, most of which have only one chiral center. Recently, researches about their potential anticancer efficacy have been carried out that β-blockers can inhibit angiogenesis and tumor cell proliferation in breast cancer, multiple myeloma, pancreatic cancer, and neuroblastoma cell lines by decreasing catecholamine-driven proliferation [1,5,6], making β-blockers jump out of the conventional field of cardiovascular disease. Therefore, there would be a large and expanding market for β-blockers, considering the high incidence of various cancer cases worldwide and that cardiovascular disease remains the most common cause of death in industrialized countries.

Nowadays, single enantiomer drugs make up a large and growing portion of over-the-counter and prescription drug products [7]. Unfortunately, most of the β-blockers are still clinically being sold as a racemic mixture except for a few of them, e.g., timolol, despite the fact that their enantiomers show significant differences in the pharmacological effects and activities [8]. As we all know, the pharmacologically inactive isomer could be inactive, possess some activity of interest, be an antagonist of the active one or have a separate activity that could be desirable or undesirable when the one enantiomer is responsible for the activity of interest [9,10]. In the case of β-blockers, the cardiac β-blocking activity generally resides in their *S* (-) enantiomer [11,12,13], and the reported *S*:*R* activity ratio ranges from 33 to 530 [14] due to the diverse degree of binding affinity to the β-receptor. For example, *S*-propranolol is 100 times more potent than *R*-propranolol [15]. Therefore, the separation of racemates of β-blockers is essential both in the laboratory and industry. Further, it is not unexpected that the single enantiomer of β-blockers has a promising prospect for development in the pharmaceutical industry.

There are plenty of approaches reported for obtaining the single enantiomer of β-blockers, which can be divided into two main classes, i.e., asymmetric synthesis and chiral separation of racemates, each with their advantages and disadvantages. Single enantiomer can be gained by the method of asymmetric synthesis at a high yield compared to the 50% yield at most for enantioseparation, while enantioseparation is quite more feasible and convenient than asymmetric synthesis with numerous separation techniques as well as chiral selectors.

When it comes to enantioseparation of β-blockers, liquid chromatography, without any doubt, has evolved over several decades and hence is one of the most mature and widely used methods up to now, far superior to classical methods, such as the formation of diastereomeric pairs followed by repeated recrystallization or the use of stereoselective enzymes. Liquid chromatography, including high performance liquid chromatography (HPLC), simulated moving bed chromatography (SMB) and so on, employ direct or indirect methods by using a great number of chiral stationary phases or chiral derivatization reagents as chiral selectors. Besides liquid chromatography, supercritical fluid chromatography (SFC), gas chromatography (GC), and thin layer chromatography (TLC) are applied to the chromatographic separation of β-blockers as well.

In this review, we focus the chromatographic methods employed to the enantioseparation of β-blockers in the 21 century, covering liquid chromatography and supercritical fluid chromatography.

## 2. Enantioseparation of β-Blockers by HPLC

In the last 30 years, HPLC has obtained a great reputation in the field of enantioseparation, owing to its rapidness, reproducibility, sensitivity, mild operating temperature and availability of a tremendous number of chiral selectors [16,17,18].

HPLC can be applied to resolve a wide range of racemic drugs, including β-blockers, either directly with chiral stationary phases (CSPs) or indirectly with chiral derivatization reagents (CDRs). Each has its advantages and disadvantages, which we will discuss later.

### 2.1. Direct Chiral Separations

An enantioselective environment must be created by applying a chiral stationary phase (CSP) or one kind of chiral mobile phase additives (CMPAs) to achieve the separation of enantiomers, which is thereby called the direct chiral separation method [19]. Therefore, it can be divided into two main groups, chiral stationary phases and chiral mobile phase additives.

Compared with CMPAs, commercial or home-made CSPs have been developing much more dramatically in the past few decades, because they require much less sample manipulation and make more rapid solute recovery possible [20]. As a result, a great deal of work has been carried out on the enantioseparation of racemic drugs by applying different varieties of CSPs, which are grouped into eight catagories, as shown in Table 3. Overviews of different separation principles and various applications of CSPs are given in [21,22,23,24,25,26].

Thanks to many years of development, there are plenty of commercial CSPs available and among them the polysaccharide type is the most successful one, including Chiralpak AD [30,31,32], IA [32], IB [33] and Chiralcel OC, OD, OF, OG, and OJ, based on amylose and cellulose, respectively. The Pirkle type, protein, β-cyclodextrin, and macrocyclic glycopeptide-based CSPs are the next four widespread CSPs [22], such as Sumichiral OA-2500, Sumichiral OA-4900 [30], R-alpha-burke1 for the Pirkle type, Chiral-CBH, Chiral-AGP, Trichsep-100, Resolvosil BSA7 for proteins, Chidex-MKP, Chidex-SKP, Ultron ES-PHCD for cyclodextrin and Chirobiotic T [30], and Chirobiotic V [34,35] for macrocyclic glycopetide-based CSPs, etc. [22].

Besides those commercial CSPs, during the last few decades, significant progresses have been made on the development of home-made CSPs used for the direct separation of β-blockers in HPLC, mainly polysaccharide derivatives [36,37,38,39,40,41], cyclodextrins [42,43,44,45,46,47], macrocyclic antibiotic [35], chiral crown ethers [48,49], and Pirkle type CSPs [50,51].

#### 2.1.1. Polysaccharide Derivatives

Chiral stationary phases based on polysaccharide derivatives showed a very broad applicability to different compound classes with high enantioselectivity. There are plenty of specialized reviews that reported the development and applications of polysaccharide-based phases [24,27,52].

Native cellulose showed only weak chiral recognition ability [53]. Then Hesse and Hagel [54] discovered that microcrystalline cellulose triacetate (CTA-I) forms chiral cavities upon swelling which are able to include stereoselectively compounds with aromatic residues. Over the years, Okamoto’s group have prepared various types of polysaccharide derivatives, coated or bonded to different supports, many of which turned out to be excellent CSPs [55,56,57]. Typical results for the enantioseparation of the β-blockers with polysaccharide based CSPs are presented in Table 4.

In 2004, Okamoto’s group first synthesized pure cellulose derivative-based beads prepared from cellulose 3,5-dimethylphenylcarbamate (CDMPC) bearing a small amount of 3-(triethoxysilyl)propyl groups, which were later utilized to join all of the CDMPC molecules together [58] and form a new type of CSPs, i.e., organic–inorganic hybrid CSPs. It is exactly because of the high loading capacity and excellent solvent tolerance of the obtained CSPs that organic-inorganic hybrid materials have attracted much attention as packing materials in HPLC [59,60].

In order to meet the requirements of mechanical strength and gain better enantioseparation ability, Weng et al. [61] prepared a hybrid cellulose 3,5-dimethylphenylcarbamate chiral stationary phase via a sol-gel method, followed by an end-capping process to shield residual silanol groups. The obtained CSP with much higher organic/inorganic ratio, good mechanical strength as well as merits of hybrid materials had better chiral recognition ability and exhibited selectivity as high as 5.55 for pindolol. Recently, a hybrid cellulose 3,5-dichlorophenylcarbamates based organic-inorganic CSP was de novo synthesized by Yu et al. through similar procedure [62]. The synthesized materials with good sphericity showed a high density of chiral selectors and good mechanical stability, while still exhibiting comparable enantioselectivity to immobilized-type and commercial Chiralpak IC.

Besides these, Mosiashvili et al. [63] systematically studied the effect of basic and acidic additives on HPLC separation of enantiomers of β-blockers on polysaccharide-based chiral columns under polar organic mobile phase conditions, which leaded to the conclusion that the presence of minor additives could bring about major changes in the resolution of enantiomers. Fu et al. [64] did some similar work and yielded the same result by evaluating the effects of adding isopropanol as organic modifier, acidic additives (formic acid), and basic additives (diethylamine) and optimizing the chiral separation method with two chiral columns (Lux Cellulose-1 and Chiralpak CBH, polysaccharide and protein-based, respectively).

#### 2.1.2. Cyclodextrin-Based CSPs

Cyclodextrin-based CSPs are also frequently used for the enantioseparation of β-blockers. Cyclodextrins (CDs), one kind of cyclic oligosaccharides, consist of six (α-CD), seven (β-CD) or eight (γ-CD) glucopyranose units. CDs possess a hydrophilic surface and a truncated cone with a hydrophobic cavity, which can be modified by derivatization on hydroxy groups in positions 2, 3 and 6. CDs’ chiral recognition mainly rely on the inclusion of the bulky hydrophobic group of the analytes into the hydrophobic cavity. Moreover, CD based CSPs can be applied in various separation modes, such as normal phase (NP), reversed phase (RP) and polar-organic phase (PO).

Over the years, plenty of researches have been done to improve the enantioselectivity or to open up the application of cyclodextrin, most of which centered around various supports for cyclodextrins to coat on or bond with, since supports also have an influence on the enantioselectivity of cyclodextrin-based CSPs.

The silica materials have been their favorite and first choice due to its characteristics, and in most cases, aminated silica gel. Poon et al. [65] immobilized the derivatized cyclodextrins onto the aminated silica gel via the Staudinger reaction and the synthetic CSPs was proved to be suitable for discriminating a wide range of structurally diverse racemic compounds, including β-blockers. To avoid the presence of free amine groups on the surface of silica gel, Lai et al. [43] chose to apply (EtO)_3_SiH as the support of cyclodextrin derivatives and it turns out that cyclodextrin CSPs obtained by this means always show better enantioresolution ability. More recently, ordered mesoporous SBA-15 was also used as supports by Zhou et al. [66] to reduce the mass transfer resistance and enhance the chiral chromatographic property of CD based CSPs and thus separate 14 kinds of β-blockers enantiomers.

Compared with supports, CDs with multiple substituents without any doubts have more possibilities and have attracted much more attention from researchers. Among all these derivatives, the most widely used and effective CSPs are aromatic derivatized CD, especially phenylcarbamoylated CDs, which can produce more interactions such as π-π interaction, dipole-dipole interaction and hydrogen bonds because of the introduction of aromatic rings, “N-H” bonds and “C=O” bonds. Jin et al. [67] synthesized phenylcarbamoylated α, β and γ-cyclodextrin chiral stationary phases and it turns out that the above CSPs have better enantioselectivity towards β-blockers than native CDs. Wang et al. [42,68] prepared two CSPs by immobilization of phenylcarbamoylated β-CDs onto alkynyl modified silica via “click” chemistry and successfully resolved β-blockers. A cholesterol mono-derivatized beta-cyclodextrin was synthesized and bonded onto SBA-15 to obtain a cholesterol mono-derivatized beta-cyclodextrin-bonded stationary phase (CHCDP) by Wang et al. [69]. The results showed that CHCDP possessed separation abilities towards β-blockers in multiple chromatographic modes.

A *N*,*N*’-ethylenediamino bridged bis(β-cyclodextrin)-bonded SBA-15 chiral stationary phase (BCDSP) for HPLC was first developed through solid successive reaction method by Zhou et al. [66] and could separate 14 kinds of β-blockers enantiomers, while native β-cyclodextrin-bonded phase with single ring ligand could partially resolve about 4 kinds of them under optimized condition. Recently, a stilbene diamido-bridged bis(β-cyclodextrin) was synthesized and bonded onto SBA-15 by Shuang et al. [70] to obtain a novel bridged bis(β-cyclodextrin)-bonded chiral stationary phase (SBCDP). Unlike the small cavity of native CD, the bridging linker of the bridged bis(β-CD) supplied a well-organized “pseudo-cavity”, and combined two native CDs as an organic whole, making the chiral discrimination of SBCDP more precise. Rahim et al. [71] prepared two covalently bonded β-CD based CSPs by immobilizing the native β-CD and β-CD-BIMOTs onto modified silica gel, and the most interesting part is that β-CD-BIMOTs is a β-CD derivatized with ionic liquid (3-benzylimidazoliumtosylate) substituent. It turns out that the enantioseparation of β-blockers with an ionic liquid moiety was better than native β-CD CSP, which proved the critical role of ionic liquid in enhancing the enantioseparation for β-blockers. Typical results for the enantioseparation of the β-blockers involving cyclodextrin based CSPs is listed in Table 5.

#### 2.1.3. Macrocyclic Antibiotics

The macrocyclic antibiotics, initiated by Armstrong in 1994 [72], such as teicoplanin, vancomycin and ristocetin A and its analogs, are also one of the most useful selectors for the enantioseparation of nonprotected and *N*-protected peptides, amino acids, β-blockers, and various biologically important compounds [73]. Their unique structural features and functionalities allow multiple chiral interactive sites and interactions between the analyte and CSPs, for example, electrostatic, hydrophobic, H-bonding, steric repulsion and so on. An additional advantage of macrocyclic antibiotics is that they can work in the NP, RP, PO and the polar ionic (PI) modes. For further details and applications, the reader is referred to specialized reviews [73,74].

Plenty of researchers [15,74,75,76,77] chose commercial chirobiotic columns, including teicoplanin containing Chirobiotic T and T2, teicoplanin aglycone containing Chirobiotic TAG, ristocetin A containing Chirobiotic R, and vancomycin containing Chirobiotic V and V2, as their chiral selector for the enantioseparation of β-blockers.

For example, enantiomeric resolution of tertatolol was achieved on a vancomycin containing Chirobiotic V chiral stationary phase with UV detection by Hefnawy et al. [35]. Bosáková et al. [34] compared the enantioseparation abilities of two vancomycin based CSPs with different coverage of the chiral selector vancomycin (Chirobiotic V and Chirobiotic V2) for β-blockers in RP and PO modes (Table 6). It turns out that higher coverage of vancomycin and polar-organic mobile phase give better enantioresolution of β-blockers. George et al. [78] went further by adopting a quality-by-design approach for enantioseparation of atenolol on vancomycin and teicoplanin-based CSPs using RP and PI mode, respectively, to account for major forces involved in enantiorecognition of β-blockers on macrocyclics. The enantioseparation of atenolol in both elution modes showed that ionic interactions governed chiral recognition on the two macrocyclic stationary phases in both modes and it was possible to establish mathematical models relating separation as well as retention parameters to the chromatographic factors studied on both modes.

Beyond that, Pérez-Quintanilla et al. [79] prepared spherical mesoporous hybrid silica materials by a post-synthesis method, modified with erythromycin and vancomycin as chiral selectors, and applied them in NP and PO modes of HPLC to resolve four β-blockers. Such hybrid materials have several advantages: better accessibility to binding sites, high adsorption capacities, better selectivity, faster adsorption rates and enhanced permeability, which will become a promising field for new investigations of macrocyclic antibiotics based CSPs.

#### 2.1.4. Crown Ethers

Crown ether-based CSPs have been believed to be effective for chiral molecules containing primary or secondary amino groups, including β-blockers, due to the enantioselective tripodal complexation of the primary ammonium ion (R-NH_3_^+^) of analytes inside the cavity of the crown ether ring of the stationary phase, which is crucial for the chiral recognition [80,81,82]. A comprehensive review on the applications of chiral crown ether to racemate was recently given by Adhikari et al. [83].

As a matter of fact, the application of chiral crown ether based CSPs to the resolution of non-primary amino compounds, including β-blockers, is much rarer compared to the above chiral stationary phases. However, there is one exception, which was successfully applied to the enantioseparation of β-blockers [84]. This special crown ether based CSP is known as (+)-(18-crown-6)-2,3,11,12-tetracarboxylic acid (Table 7). Steffeck and co-workers [84] applied it for the resolution of three β-blockers with the use of a polar organic mobile phase, and the resolutions (Rs) were found to vary from 1.16 for atenolol to 2.38 for propranolol. Afterwards, Zhang et al. [85] followed the Former’s steps and successfully employed this CSP in the resolution of eleven β-blockers with the use of an optimized mobile phase, and the Rs were in the range of 1.36–5.79. Interestingly, the separation factors were observed to increase as the temperature increased, which was in contrast to the resolution of primary amino compounds.

Besides (+)-(18-crown-6)-2,3,11,12-tetracarboxylic acid, Hirose et al. [48] prepared chiral crown ether (*S*,*S*)-3 having a pseudo-24-crown-8 ring and chiral podand (*R*,*R*)-4, which exhibited good chiral recognition ability toward propranolol.

#### 2.1.5. Protein-Based CSPs

Protein-based CSPs are the first introduced chiral stationary phases and were once very popular. However, now, they are less and less used due to their fatal drawbacks of limited capacity and fragility [86]. Still, there are several commercial CSPs based on protein, which are quite successful for the enantioseparation of β-blockers, i.e., Chiral-CBH, Chiral-AGP, EnantioPac, Resolvosil, and Ultron-ES-OVM [22] (Table 8). Recently, six β-blockers enantiomers were separated on ovomucoid (OM) column in optimal conditions by Imre and co-workers [87]. They also investigated the effect of the organic modifier, the influence of pH and the percentage of the aqueous phase on resolution. It was proved that OM was suitable for enantiomeric separation of four nonselective β-blockers, in contrast with the two β-1 selective blockers, atenolol and metoprolol.

In addition to commercial columns, Matsunaga et al. [88] immobilized cellulase onto aminopropyl-silica gels via its amino and carboxy groups, respectively, and the nominal particle diameters of silica gel were 5, 3, and 2.1 μm. It turns out that the method of immobilization had little impact on the resolution ability of the cellulase-based CSPs. In addition, the CSP prepared with 2.1 μm aminopropyl-silica gels gave the highest enantioselectivity and column efficiency among all three columns. Furthermore, the results indicated that the enantioselective bindings of β-blockers could occur at the catalytic binding site and the secondary binding site. Typical results are presented in Table 9.

Besides the above CSPs, Haginaka and Sakai [89] synthesized a uniform-sized molecularly imprinted polymer material using methacrylic acid and ethylene glycol dimethacrylate, respectively, as a host functional monomer and cross-linker, which had specific recognition for *S*-propranolol and related β-blockers. An amide based chiral stationary phase m-[(+)-alpha-methyl benzyl carboxamide] XAD-4 has been synthesized by covalently linking *R-*(+)-1-phenylethylamine to chloroformoyl Amberlite XAD-4 by Agrawal and Patel [90], which as a result was the functionalized polymer beads with porous structures.

### 2.2. Indirect Separation Methods

Unlike the direct one, the indirect separation methods use optically pure CDRs to form diastereomers, differ in their physical and chemical properties, and thus they can be resolved by achiral chromatography [8,91].

This approach reduces the analysis cost via using inexpensive achiral columns rather than expensive chiral columns, and the chromatographic conditions can be optimized much more conveniently as CDRs provide a highly sensitive detector respondence which may lead to better resolution [92]. However, the indirect method also has some severe disadvantages compared to the direct one. The derivatization process is quite time-consuming and laborious, and puts high demand on the purity of CDRs, let alone the possibilities of side reactions. Therefore, researches about the indirect one are relatively less, and furthermore mainly focus on the study of CDRs. The ideal CDR should be accessible, inexpensive, and easy to form diastereomers which are optically stable under common conditions.

Researchers prefer CDRs which can form C-N bond by reacting with the secondary amines of β-blockers. Up to now, the frequently used CDRs can be divided into four main classes, i.e., cyanuric chloride (CC) based CDRs (including MCT and DCT), naproxen based CDRs, Marfey’s reagent based CDRs and isothiocyanates. Typical CDRs applied to the enantioseparation of β-blockers are presented in Table 10, while typical results are listed in Table 11.

Cyanuric chloride (CC; trichloro-*S*-triazine; *S*-triazine chloride; 2,4,6-trichloro-1,3,5- triazine) has the prospect of easy and sequential substitution of its chlorine atoms with nucleophiles by controlling the reaction conditions appropriately [93]. Further, the sequential and controlled substitution of chlorine atom(s) by nucleophiles in CC provides monochloro-*S*-triazine (MCT) and dichloro-*S*-triazine (DCT) reagents, which were used for the enantioresolution of several primary or secondary amino group containing analytes, e.g., β-blockers. Bhushan et al. [94] synthesized 15 CDRs on the cyanuric chloride platform and successfully resolve five β-blockers through C_18_ column by RP-HPLC.

**Figure 1:**
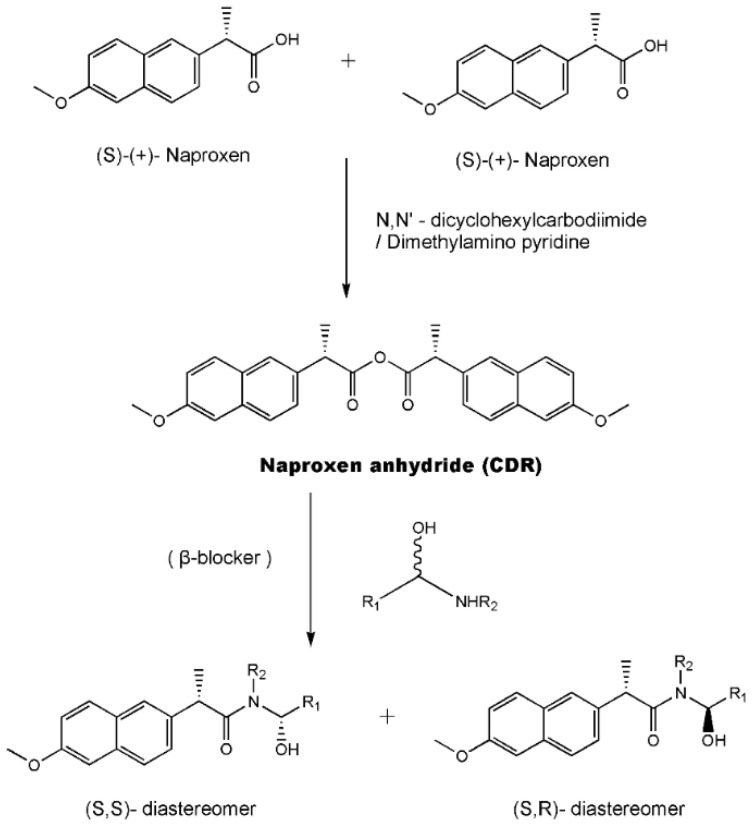
The preparation procedure of naproxen anhydride (CDR) and formation of diastereomers of β-blockers [95].

As one kind of non-steroidal anti-inflammatory drugs, naproxen (*S*-2- (6-methoxynaphthalen-2-yl) propanoic acid) has also been used in HPLC for its characteristic features of having chiral center with high molar absorptivity. Recently, an anhydride type CDR was synthesized from *S*-(+)-naproxen by Bhushan et al. [95], presented in Figure 1, and was used for C-N bond formation to prepare diastereomeric amides of *RS*-propranolol, *RS*-atenolol, *RS*-carvedilol and *RS*-metoprolol. Marfey’s reagent, namely, 1-fluoro-2,4-dinitrophenyl-l-alaninamide (FDNP-l-Ala-NH_2_), was first prepared via the substitution of one fluorine atom in DFDNB (Sanger’s reagent, namely, 1,5-difluoro-2,4-dinitrobenzene) with l-alanine by Marfey in 1984 [96]. It was used as a chiral derivatization reagent for the enantioseparation of amino acid, and now of β-blockers. Bhushan and his team have done a lot in this area. In 2009, Bhushan et al. [92] synthesized twelve CDRs based on Sanger’s reagent and subsequently six β-blockers were separated under reversed phase conditions by HPLC. Two years later, three kinds of Marfey’s reagent were also prepared by introducing two chirally pure amines and one L-amino acid via substitution of one fluorine atom in DFDNB [97] and successfully obtained diasteromers of betaxolol and propranolol.

**Figure 2:**
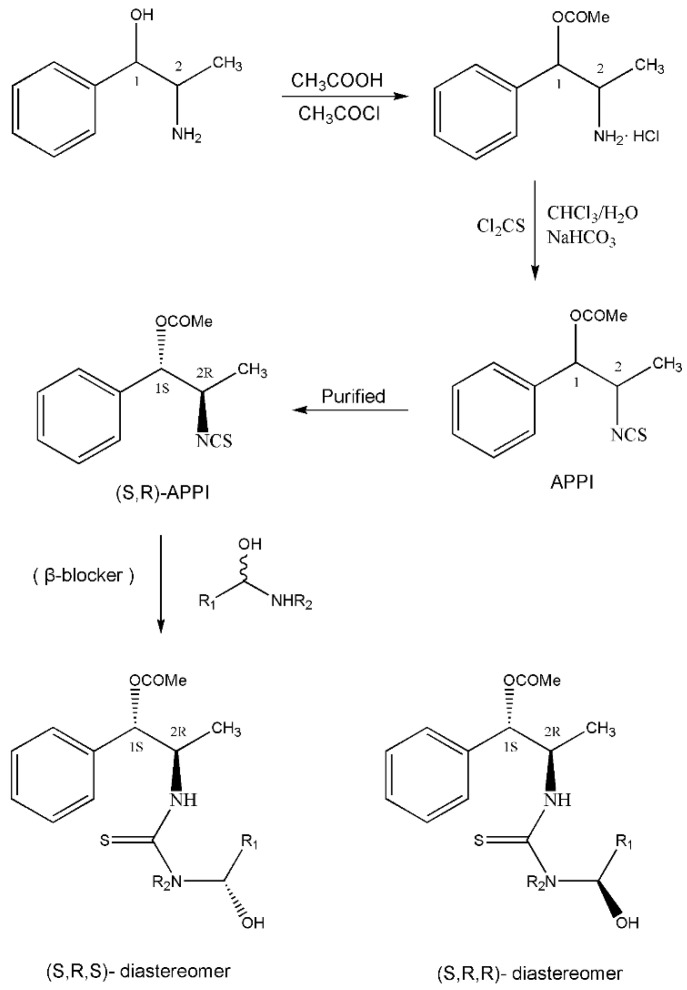
The preparation procedure of (S,R)-APPI and synthesis of diastereomers of β-blockers [98].

**Figure 3:**
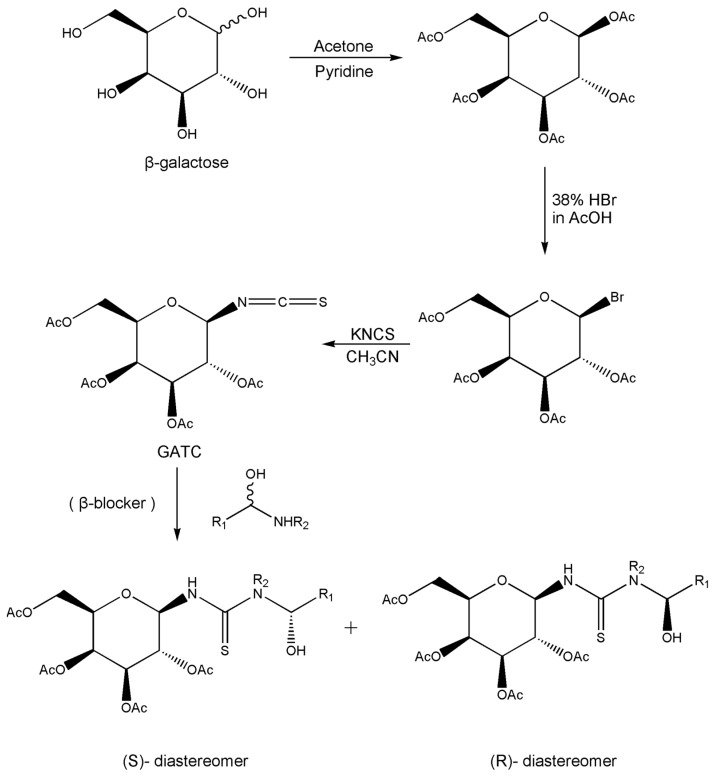
Scheme of the synthesis route of GATC and formation of diastereomers of β-blockers [99].

In addition to the above materials, several isothiocynates, with their typical structure features, are also quite popular for the enantioseparation of β-blockers through indirect methods. 1*S*,2*R*-1-acetoxy-1-phenyl-2-propyl isothiocyanate (*S*,*R*-APPI) was synthesized by Péter et al. [98] starting from norephedrine (Figure 2), which was stable without decomposition for several months. Ko et al. [99] reported one chiral derivatization reagent, 2,3,4,6-tetra-*O*-acetyl-β-d-galactopyranosyl isothiocynate (GATC), similar to another popular isothiocyanate based CDR, GITC, and subsequently optimized the derivatization and chromatographic conditions for its application of β-blockers in RP-HPLC. The scheme of the synthesis route of GATC and their diastereomers of β-blockers is presented in Figure 3.

Beyond that, Sung et al. [100] applied *S*-(-)-menthyl chloroformate((-)-MCF) as the chiral derivatization reagent, combined with hydrolysis, to resolve stereoisomers of nadolol with three chiral centers and finally obtained the most active one *R,S,R*-(-)-nadolol with very good optic purity (99.97%).

## 3. Enantioseparation of β-Blockers by SFC

In recent years, the rapidly emerging technique of SFC, one kind of chromatography with a sub- or supercritical fluid (i.e., carbon dioxide) as mobile phases, has reached a new level of utility in the enantioseparation of racemic drugs [101,102,103,104,105,106,107].

As a bridge between GC and LC, SFC has several significant advantages over them. Compared to GC, the ability to resolve thermally labile and nonvolatile analytes is the greatest strength of SFC. Compared to HPLC, SFC often provides higher resolution capacities and needs shorter equilibrium times, along with lower consumption of organic solvents [106], since supercritical fluids possess special characteristics of lower viscosity, higher diffusivity and favorable transfer characteristics than normal mobile phases. By the way, these unique merits of supercritical fluids of SFC also make it possible to scale up as well as prepare kilograms of materials, and now SFC is becoming the primary method for preparative chiral chromatography. In addition, HPLC and GC detectors are both compatible with SFC and a majority of CSPs developed for LC is suitable with SFC too, except a few, i.e., immobilized chiral protein-based or crown-ether stationary phases [108]. However, SFC also has the disadvantage of requiring special equipments, such as a chilled pump, a restrictor and pressure regulator [109]. There are also some specific reviews of SFC for a more particular knowledge of this area [110,111].

In chiral SFC of β-blockers, the majority of separations are still performed by the direct approach, just like in HPLC, which can be divided into two main classes, i.e., CSPs and CMPAs. Typical results are presented in Table 12.

Firstly, polysaccharide and macrocyclic antibiotic based CSPs appear, by far, as the most successful and broadly applied phases in enantioseparation of β-blockers by SFC. There are plenty of articles that reported the resolution of several specific β-blockers on commercial chiral columns based on polysaccharides and macrocyclic antibiotics, such as Chiralpak AD [101,112,113], Chiralcel OD [101,103,112], Chirobiotic R [114], Chirobiotic V [115] and Chirobiotic TAG [114], etc.

In addition to these commercial columns, Kalíková et al. [116] applied cellulose tris-(3,5-dimethylphenylcarbamate) as the chiral stationary phases on the enantioseparation of β-blockers in SFC. The effect of both mobile phase co-solvents (MeOH or PrOH) and mobile phase additives (IPA, DEA, TEA, TFA, or TFA/IPA) on the enantioselective separation was also studied. Kraml and co-workers [103] found that the carbobenzyloxy (cbz) protecting group was able to enhance the resolution of β-blocker enantiomers on both polysaccharide and Pirkle-type columns under the supercritical fluid chromatography condition. The effect of increasing the concentration of 2-propylamine as additive on the elution of a series of basic compounds, including β-blockers on a Chiralpak-AD stationary phase was studied by Speybrouck et al. [117]. It turns out that all β-blockers generated inverted U shape curves for the selectivity factor vs additive concentration. Recently, a multi-residue method by SFC coupled with tandem mass spectrometry method were adopted by Rice et al. [118] for the analysis and enantiosepration of 140 chiral and non-chiral chemicals in environmental samples, including β-blockers, using 2.1 μm particle size. It turns out that SFC is an excellent technique for combined achiral-chiral analysis due to the combined use of supercritical CO_2_, non-biological chiral selectors, and smaller-UHPLC-size particles.

Secondly, there are less researches about the application of CMPAs on β-blockers in SFC compared with CSPs. Gyllenhaal et al. [105] once used l-(+)-tartaric acid as a CMPA in a packed-column SFC system. It turns out that l-(+)-tartaric acid can be used to generate an enantioselective chromatographic system together with Hypercarb as support and methanol-modified carbon dioxide as the mobile phase with an appropriate amine additive and increased concentrations of CMPA gave higher retention and also increased the enantioselectivity.

## 4. Enantioseparation of β-Blockers by SMB

SMB is not only a special technique for preparative liquid chromatography, but also one of the most promising methods for the enantioseparation of racemic drugs with several chiral centers after the first successful enantioseparation on SMB by Negawa and Shoji [119] in 1992. SMB is feasible at all production scales, from laboratory to production plant [120]. As a mature technology, SMB also have several advantages over other preparative technology. Since the process is continuous, SMB enable unattended operation and stable product quality and it can exhibit similar performance with lower solvent consumption, high productivity and less stationary phases [120]. It is also worth noting that SMB has one major disadvantage compared to batch preparative chromatography, that SMB generally can only produce two fractions, one in the raffinate and the other in the extract stream, although which is especially suitable for enatioseparation with one chiral center. A typical SMB system, in most cases divided into four or five zones, always consists of an array of columns connected in series (commonly 6–12 columns), valves and several pumps including at least one recycling pump for delivering the mobile phase flowing through all columns [91]. The primary character of SMB is the continuous countercurrent movement of the stationary and mobile phases [121] where the simulated movement is created by periodic switching of injection and withdrawal ports along the columns in direction of fluid flow [122].

Despite all the mentioned advantages, the application of SMB to chirotechnology, especially to the enantioseparation of β-blockers, has been quite rare in recent years, and the biggest challenge lies in finding the perfect CSPs. The word perfect means CSPs applied in SMB should have the common features of the excellent CSPs in HPLC, such as good selectivity, wonderful mechanical and chemical stability, long lifetime, and so on. Nevertheless, the prerequisite for the scale-up of a chromatographic chiral separation is that the CSP is available in large amounts, with reproducible batch-to-batch properties and at relatively low cost with respect to the value of the enantiomers to be separated [120].

Given the large amount of CSPs used in SMB units, cheap materials, such as cyclodextrins and polysaccharides, are always the most suitable chiral stationary phases for the resolution of racemic drugs. Wang and Ching have put in a lot of effort to study the application of the perphenyl carbamoylated β-cyclodextrin in the resolution of nadolol, the three-chiral-center β-blocker drug, using SMB. They used the h-root method to determine the competitive Langmuir isotherm for stereoisomers of nadolol on a perphenyl carbamoylated β-cyclodextrin bonded chiral stationary phase [123]. In 2004, they applied the four-zone open-looped SMB separation unit, which consisted of eight columns arranged in a 2-2-2-2 configuration. After several runs, the product purity of extract and raffinate can reach 99.4% and 99.8%, respectively. A direct simulation approach has also been proposed to simulate the SMB separation performance for the pseudo-binary mixture of nadolol [124]. Greater progress was made by Wang’s group in the enantioseparation of nadolol by applying 2-raffinate or 2-extract five-zone SMB instead of four in the next year [125]. Separation performances of the five-zone SMB were investigated for both 2-raffinate and 2-extract configurations.

As for polysaccharide-based CSPs, Ribeiro et al. [126] separated the four nadolol stereoisomers on Chiralpak AD at both analytical and preparative scales. Particularly, this work presents the experimental separation of the more retained nadolol stereoisomer (*RSR*-nadolol) by SMB using an 80:20:0.3 ethanol-heptane-diethylamine mobile phase. The pseudo binary separation of the nadolol stereoisomers was also performed using Chiralpak IA in SMB by Arafah et al. recently [127]. The more retained component was fully recovered with 100% purity and 100% recovery.

Besides cyclodextrins and polysaccharides, protein-based CSPs were also used in enantioresolution of β-blockers in SMB. Zhang et al. [128,129] have made considerable efforts on the enantiosepartion of pindolol on α_1_-acid glycoprotein chiral stationary phases by SMB. The effects of column configurations and the isotherm parameters on system performance were also investigated for SMB operation [122]. Results demonstrate that column configuration with 1/2/1/1 is superior to that with 1/1/2/1 in achieving higher purity of both raffinate and extract and decrease of saturation capacity of either site will cause a decrease in raffinate recovery.

## 5. Enantioseparation of Propranolol

As described above, within the last few decades, a large number of investigations on the development of new enantioselective methods has been carried out in the field of β-blockers, for instance, HPLC, SFC and SMB. In this section, we take a specific example of β-blocker, i.e., propranolol, which was the first β-blocker used in clinic and is still clinically applied, to illustrate the pros and cons of enantioseparation of β-blockers. Table 13 shows three methods, i.e., CSPs, CDRs, and SFC, applying for the enantioseparation of propranolol, making it quite obvious the pros and cons of each approach. Firstly, the enantioresolution of β-blockers via CSPs is without doubt the most mature and popular technology nowadays, with plenty of CSPs available but also at the cost of comparatively lower resolution (2.0 to 7.04 versus 7.5 to 14.12 for propranolol). Secondly, the approach of CDRs reduces the analysis cost via using inexpensive achiral columns and provides a highly sensitive detector respondence and better resolution, at the meantime, imposing an additional strain on the purity of CDRs and the synthesis of diastereomers of β-blockers. Researches about the resolution of β-blockers by SFC are relatively less, compared to the other methods. Futhermore, there are even fewer about the resolution of β-blockers by SMB, since SMB is mainly applied to sample preparation, or commercial production. In principle, most CSPs developed for LC could be applied both in SFC and SMB, while shorter equilibrium times could usually be obtained in SFC.

## 6. Conclusions

β-blockers are still an important class of drugs in the market. At least 70 β-blockers have been investigated in history. Only a few β-blockers, e.g., timolol, are clinically marketed as an optically pure enantiomer. Therefore, the separation of racemates of β-blockers is essential both in the laboratory and industry. Many approaches have been reported to separate the enantiomers. Most of them use HPLC, SFC and SMB with CSPs. The key factor is chiral selectors, whatever the approach. Many CSPs have been developed commercially or home-made. The main classes of CSPs used in the enantioseparation field can also be used in the resolution of β-blockers. New CSPs with a universal chiral recognition ability, high loading capacity, low cost, and new methods are still needed from the viewpoint of preparative scale separation. Miniaturization will also remain a trend in the future from the viewpoint of analytical scale separation.

## Figures and Tables

**Table 1:** Chemical structures of typical β-blockers with different chiral centers.

Name	Chemical Structure
Atenol	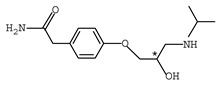
Carvedilol	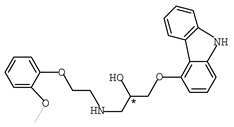
Metoprolol	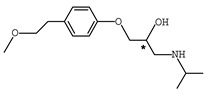
Oxprenolol	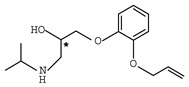
Propranolol	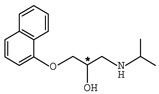
Sotalol	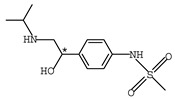
Timolol	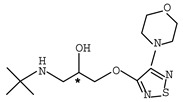
Labetalol	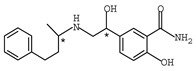
Nadolol	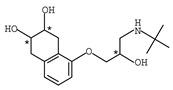
Nebivolol	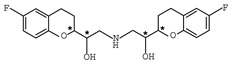

* indicates chiral center.

**Table 2:** List of β-blockers reported in the literature.

Name	CAS No.	Molecular Formula	No. of Chiral Center	Name	CAS No.	Molecular Formula	No. of Chiral Center
Acebutolol	37517-30-9	C_18_H_28_N_2_O_4_	1	Idropranolol	27581-02-8	C_16_H_23_NO_2_	1
Adimolol	78459-19-5	C_25_H_29_N_3_O_3_	1	Indenolol	60607-68-3	C_15_H_21_NO_2_	1
Afurolol	65776-67-2	C_15_H_21_NO_4_	1	Labetalol	36894-69-6	C_19_H_24_N_2_O_3_	2
Alprenolol	13655-52-2	C_15_H_23_NO_2_	1	Landiolol	133242-30-5	C_25_H_39_N_3_O_8_	2
Amosulalol	85320-68-9	C_18_H_24_N_2_O_5_S	1	Mepindolol	23694-81-7	C_15_H_22_N_2_O_2_	1
Ancarolol	75748-50-4	C_18_H_24_N_2_O_4_	1	Metipranolol	22664-55-7	C_17_H_27_NO_4_	1
Arnolol	87129-71-3	C_14_H_23_NO_3_	1	Metoprolol	37350-58-6	C_15_H_25_NO_3_	1
Arotinolol	68377-92-4	C_15_H_21_N_3_O_2_S_3_	1	Moprolol	5741-22-0	C_13_H_21_NO_3_	1
Atenolol	29122-68-7	C_14_H_22_N_2_O_3_	1	Nadolol	42200-33-9	C_17_H_27_NO_4_	3
Befunolol	39552-01-7	C_16_H_21_NO_4_	1	Nebivolol	99200-09-6	C_22_H_25_F_2_NO_4_	4
Betaxolol	63659-18-7	C_18_H_29_NO_3_	1	Nifenalol	7413-36-7	C_11_H_16_N_2_O_3_	1
Bevantolol	59170-23-9	C_20_H_27_NO_4_	1	Nipradilol	81486-22-8	C_15_H_22_N_2_O_6_	2
Bisoprolol	66722-44-9	C_18_H_31_NO_4_	1	Oxprenolol	6452-71-7	C_15_H_23_NO_3_	1
Bopindolol	62658-63-3	C_23_H_28_N_2_O_3_	1	Pafenolol	75949-61-0	C_18_H_31_N_3_O_3_	1
Bornaprolol	66451-06-7	C_19_H_29_NO_2_	3	Pamatolol	59110-35-9	C_16_H_26_N_2_O_4_	1
Brefanolol	104051-20-9	C_22_H_28_N_2_O_2_	1	Pargolol	47082-97-3	C_16_H_23_NO_3_	1
Bucindolol	71119-11-4	C_22_H_25_N_3_O_2_	1	Penbutolol	36507-48-9	C_18_H_29_NO_2_	1
Bucumolol	58409-59-9	C_17_H_23_NO_4_	1	Penirolol	58503-83-6	C_15_H_22_N_2_O_2_	1
Bufetolol	53684-49-4	C_18_H_29_NO_4_	2	Pindolol	13523-86-9	C_14_H_20_N_2_O_2_	1
Bufuralol	54340-62-4	C_16_H_23_NO_2_	1	Pirepolol	69479-26-1	C_21_H_32_N_4_O_5_	1
Bunitrolol	34915-68-9	C_14_H_20_N_2_O_2_	1	Practolol	6673-35-4	C_14_H_22_N_2_O_3_	1
Bunolol	27591-01-1	C_17_H_25_NO_3_	1	Procinolol	27325-36-6	C_15_H_23_NO_2_	1
Bupranolol	14556-46-8	C_14_H_22_ClNO_2_	1	Pronetalol	54-80-8	C_15_H_19_NO	1
Butofilolol	64552-17-6	C_17_H_26_FNO_3_	1	Propranolol	525-66-6	C_16_H_21_NO_2_	1
Carazolol	57775-29-8	C_18_H_22_N_2_O_2_	1	Soquinolol	61563-18-6	C_17_H_26_N_2_O_3_	1
Carteolol	51781-06-7	C_16_H_24_N_2_O_3_	1	Sotalol	3930-20-9	C_12_H_20_N_2_O_3_S	1
Carvedilol	72956-09-3	C_24_H_26_N_2_O_4_	1	Talinolol	57460-41-0	C_20_H_33_N_3_O_3_	1
Celiprolol	56980-93-9	C_20_H_33_N_3_O_4_	1	Tertatolol	34784-64-0	C_16_H_25_NO_2_S	1
Cetamolol	34919-98-7	C_16_H_26_N_2_O_4_	1	Tilisolol	85136-71-6	C_17_H_24_N_2_O_3_	1
Cicloprolol	63659-12-1	C_18_H_29_NO_4_	1	Timolol	29023-48-1	C_13_H_24_N_4_O_3_S	1
Cloranolol	39563-28-5	C_13_H_19_Cl_2_NO_2_	1	Tiprenolol	26481-51-6	C_13_H_21_NO_2_S	1
Epanolol	86880-51-5	C_20_H_23_N_3_O_4_	1	Tolamolol	38103-61-6	C_19_H_24_N_2_O_4_	1
Esmolol	81147-92-4	C_16_H_25_NO_4_	1	Toliprolol	2933-94-0	C_13_H_21_NO_2_	1
Exaprolol	55837-19-9	C_18_H_29_NO_2_	1	Xamoterol	81801-12-9	C_16_H_25_N_3_O_5_	1
Flestolol	87721-62-8	C_15_H_22_FN_3_O_4_	1	Xibenolol	81584-06-7	C_15_H_25_NO_2_	1

**Table 3:** Types of CSPs and their general recognition mechanisms with loading capacity [20,27,28,29].

Type	CSPs	General Recognition Mechanisms	Common Loading Capacity (mg Solute/g CSP)
1	Pirkle-type	π-π interactions; dipole stacking	1–50
2	Polysaccharide derivatives	Hydrogen bonding; inclusion of a chiral cavity	1–150
3	β-cyclodextrins	Hydrogen bonding; dipole-dipole interactions at the mouth of CD	0.1–5
4	Macrocyclic glycopeptides	Dipole stacking; steric repulsions; π-π interactions	0.1–5
5	Chiral crown ether	Forming an inclusion complex between the primary amine and the chiral crown ether	0.1–5
6	Ligand exchange	Forming bidentate chelates	0.1–1
7	Proteins	Hydrophobicity; hydrogen bonds; electrostatic interactions	0.1–0.2
8	Other polymers		1–100

**Table 4:** Typical results for the enantioseparation of the β-blockers with polysaccharide based CSPs.

CSP	Drug	Mobile Phase (*v*/*v*)	T (℃)	Flow-Rate (mL/min)	First	α	Rs	Ref.
CDMPC-silica hybrid spheres	Pindolol	*n*-Hexane/1-Propanol/DEA (80/20/0.5)	30	1.0	/	5.55	5.95	[61]
ADMPC	Carvedilol	IPA/DEA (100/0.1)	20	1.0	S	/	/	[63]
Lux Cellulose-1	Carvedilol	ACN/DEA (100/0.1)	20	1.0	S	/	/	[63]
Lux Cellulose-1	Propranolol	IPA/DEA/FA (100/0.1/0.1)	20	1.0	R	/	/	[63]
Lux Cellulose-1	Metoprolol	ACN/IPA/DEA/FA (80/20/0.1/0.1)	30	0.1	/	2.9	8.9	[64]
Chiralpak CBH	Atenolol	(1 mM NH_4_Ac aqueous)/IPA (90/10)	30	0.1	R	2.05	18.7	[64]

DEA: Diethylamine; IPA: 2-Propanol; ACN: Acetonitrile; FA: Formic acid.

**Table 5:** Typical results for the enantioseparation of the β-blockers with cyclodextrin based CSPs.

CSP	Drug	Mobile Phase (*v*/*v*)	T (℃)	Flow-Rate (mL/min)	α	Rs	Ref.
Mono-2^A^-azido-2^A^-deoxyperphenyl-carbamoylated β-CD	Alprenolol	Buffer (1% TEA, pH 5.11)/MeOH (70/30)	23	1.0	2.02	1.25	[65]
Mono(6^A^ -*N*-allylamino-6^A^ -deoxy) perphenyl-carbamoylated β-CD	Propranolol	Buffer (1% TEA, pH 5.50)/ MeOH (75/25)	23	0.5	1.51	4.7	[43]
BCDSP	Esmolol	ACN/MeOH/TEA/ HOAc (95/5/1/1)	15	1.0	1.24	1.59	[66]
BCDSP	Arotinolol	ACN/MeOH/TEA/ HOAc (85/15/1/1)	15	1.0	1.23	1.58	[66]
Perphenylcarbamated CD	Nadolol	(1%TEAA, pH 6.5)/MeOH (70/30)	/	0.7	1.35	1.99	[68]
CHCDP	Atenolol	ACN/MeOH/TEA/ HOAc (96/4/1/1)	25	0.5	1.11	1.57	[69]
β-CD-BIMOTs	Metoprolol	ACN/Water (90/10)	30	0.5	1.78	2.38	[71]

TEA: Triethylamine; MeOH: Methanol; HOAc: Acetic acid; TEAA: Triethylamine acetate.

**Table 6:** Typical results for the enantioseparation of β-blockers on Chirobiotic V and Chirobiotic V2.

CSP	Drug	Mobile Phase (*v*/*v*)	T (℃)	Flow-Rate (mL/min)	α	Rs	Ref.
Chirobiotic V	Tertatolol	MeOH/HOAc/TEA (100/0.01/0.015)	/	0.8	1.39	1.9	[35]
Chirobiotic V	Propranolol	MeOH/(0.5%TEAA, pH 5.0) (90/10)	22	1.0	1.12	2.0	[34]
Chirobiotic V	Acebutolol	MeOH/(0.5%TEAA, pH 5.0) (90/10)	22	1.0	1.11	1.68	[34]
Chirobiotic V2	Alprenolol	MeOH/HOAc/TEA (100/0.01/0.01)	22	2.0	1.12	2.06	[34]
Chirobiotic V2	Pindolol	MeOH/HOAc/TEA (100/0.01/0.01)	22	2.0	1.13	2.14	[34]

**Table 7:** Typical results for the enantioseparation of β-blockers on the (+)-(18-crown-6)-2,3,11,12-tetracarboxylic acid based CSP.

CSP	Drug	Mobile Phase (*v*/*v*)	T (℃)	Flow-Rate (mL/min)	α	Rs	Ref.
(+)-(18-crown-6) -2,3,11,12-tetracarboxylic acid based CSP	Pindolol	MeOH/ACN/HOAc/TEA (50/50/0.1/0.1)	25	1.0	1.14	2.29	[84]
	Propranolol	MeOH /ACN/ HOAc/TEA (50/50/0.1/0.1)	25	1.0	1.15	2.38	[84]
	Propranolol	ACN/ Ethanol /TEA/TFA (80/20/0.5/0.1)	40	1.0	1.30	3.43	[85]
	Oxprenolol	ACN/ MeOH/TEA (80/20/0.5/0.1)	20	1.0	1.21	2.69	[85]
	Acebutolol	ACN/ MeOH/TEA/TFA (80/20/0.5/0.1)	20	1.0	1.25	2.94	[85]

TFA: Trifluoroacetic acid.

**Table 8:** Types of protein-based CSPs for the enantioseparation of β-blockers [22].

Trade Name	Selector	Analytes
Chiral-AGP/ EnantioPac	α_1_-acid glycoprotein	Aptol, Bev, Buni, Buno, Bup, Cara, Carv, Cel, Mep, Met, Nad, Oxp, Pen, Pin, Pro, Ter, Tim, Tip, Toli
Chiral-CBH/ TrichSep-100	Cellobiohydrolase	Acecor, Aptol, Bet, Bis, Epanolol, Met, Mop, Oxp, Pam, Pra, Pro, Tal, Tim, Tol, Toli
Ultron-ES-OVM	Ovomucoid	Aptol, Nad, Pin, Pro
Resolvosil BSA	Bovine serum albumin	Bop

Acecor: Acebutolol; Aptol: Alprenolol; Bet: Betaxolol; Bev: Bevantolol; Bis: Bisoprolol; Bop: Bopindolol; Buni: Bunitrolol; Buno, Bunolol; Bup: Bupranolol; Cara: Carazolol; Carv: Carvedilol; Cel: Celiprolol; Mep: Mepindolol; Met: Metoprolol; Mop: Moprolol; Nad: Nadolol; Oxp: Oxprenolol; Pam: Pamatolol; Pen: Penbutolol; Pin: Pindolol; Pra: Practolol; Pro: Propranolol; Tal: Talinolol; Ter: Tertatolol; Tim: Timolol; Tip: Tiprenolol; Tola: Tolamolol; Toli: Toliprolol.

**Table 9:** Typical results for the enantioseparation of β-blockers on Protein-based CSPs.

CSP	Drug	Mobile Phase (*v*/*v*)	T (℃)	Flow-Rate (mL/min)	α	Rs	Ref.
Ovomucoid	Carvedilol	(20 mM KH_2_PO_4_, pH 6.7)/ACN (77/23)	25	1.0	/	2.15	[87]
	Propranolol	(20 mM KH_2_PO_4_, pH 6.7)/ACN (75/25)	25	1.0	/	1.89	[87]
	Oxprenolol	(20 mM KH_2_PO_4_, pH 6.7)/ACN (83/17)	25	1.0	/	1.95	[87]
	Pindolol	(20 mM KH_2_PO_4_, pH 6.7)/ACN (85/15)	25	1.0	/	1.75	[87]
Cellulase immobilized via amino groups	Alprenolol	(20 mM NaH_2_PO_4_, pH 6.8)/IPA (95/5)	25	0.2	6.57	8.03	[88]
Cellulase immobilized via carboxy groups	Propranolol	(20 mM NaH_2_PO_4_, pH 6.8)/IPA (95/5)	25	0.2	4.58	7.04	[88]

**Table 10:** Typical chiral derivatization reagents applied to the enantioseparation of β-blockers.

CDR	Structure	
CC based CDRs	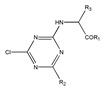	R_1_ = -OH, -NH_2_ R_2_ = -Cl, -OCH_3_, -NHCH(CH_2_C_6_H_5_)CONH_2_ R_3_ = -CH_3_, -C_6_H_5_, -CH(CH_3_)_2_, -(CH_2_)_2_SCH_3_, -CH_2_CH(CH_3_)_2_
Naproxen based CDRs	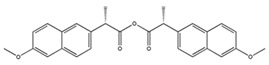	/
Marfey’s reagent based CDRs	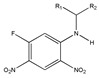	R_1_= -COOH, -CH_3_ R_2_= -CH_2_SCH_3_, -C_6_H_11_, -C_6_H_5_
	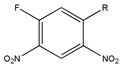	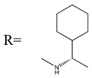
		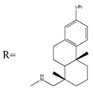
		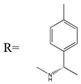
	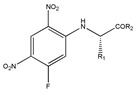	R_1_= -CH_3_, -C_6_H_5_, -CH_2_Ph, -CH(CH_3_)_2_, -CH_2_CH(CH_3_)_2_, -CH_2_CH_2_SCH_3_ R_2_= -NH_2_, -OH
Isothiocyanates	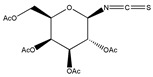	GATC
	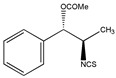	APPI
	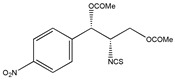	(*S*,*S*)-DANI
Others	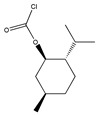	(-)-MCF

**Table 11:** Typical results for the enantioseparation of β-blockers involving CDRs.

CDR	Drug	Column	Mobile Phase (*v*/*v*)	T (℃)	Flow-Rate (mL/min)	α	Rs	Ref.
*N*-(4,6-Dichloro-[1,3,5]triazine-2-yl)-l-Val	Propranolol	LiChrospher C18	Eluent A: H_2_O/MeCN/TFA (90/10/0.1); B: H_2_O/MeCN/TFA (80/20/0.1)	/	1.0	1.10	9.83	[94]
*N*-(4,6-Dichloro-[1,3,5]triazine-2-yl)-l-Val-NH_2_	Bisoprolol			/	1.0	1.06	10.24	[94]
FDNP-l-Leu-NH_2_	Atenolol	Eurospher C18	Eluent A: MeCN; Eluent B: TFA/CH_3_CN	/	1.0	1.32	11.21	[92]
(S)-(−)-α,4-dimethylbenzylamine substituted DFDNB	Propranolol			/	1.0	1.07	10.07	[92]
*N*-succinimidyl-(S)-2-(6-methoxynaphth-2-yl) propionate	Metoprolol	Eurospher C18	Eluent A: TEAP (50 mM, pH 4.0)/CH_3_CN (70/30); Eluent B: TEAP (50 mM, pH 4.0)/CH_3_CN (30/70)	/	1.0	1.38	7.78	[92]
(R)-(−)-1-cyclohexylethylamine substituted DFDNB	Carvedilol	Eurospher C18	Eluent A: MeCN; Eluent B: TFA/CH_3_CN	/	1.0	1.05	3.28	[92]
(S)-*N*-(1-cyclohexyl-ethyl)-5-fluoro-2,4-dinitrobenzenamine	Betaxolol	LiChrospher C18	Linear gradient (35 to 65%) of ACN with 0.10% TFA	25	1.0	1.06	9.65	[97]
(S,R)-APPI	Atenolol	Nova-Pak C18	MeOH/H_2_O (60/40)	/	/	1.73	3.16	[98]
GATC	Acebutolol	Mightysil RP-18 GP column	(50 mM NH_4_OAc buffer, pH 6.0) /ACN (50/50)	RT	3.0	1.16	3.37	[99]
GITC	Bevantolol			RT	3	1.15	3.99	[99]

TEAP: Tetraethylammonium perchlorate; NH_4_OAc: Ammonium acetate; RT: Room temperature.

**Table 12:** Typical results for the enantioseparation of β-blockers in SFC.

Column	Drug	Mobile Phase (*v*/*v*)	T (℃)	BP (MPa)	Flow-Rate (mL/min)	α	Rs	Ref.
Cellulose-SBS	Alprenolol	CO_2_/PrOH/IPA/TFA (80/20/0.05/0.05)	35	13.79	2.5	/	/	[116]
Chiralpak AS	cbz-Acebutalol	CO_2_/IPA/DEA (80/20/0.1)	40	/	2.5	1.34	7.26	[103]
Chiralpak AD-H	Propranolol	CO_2_/IPA/DEA (70/30/0.1)	40	/	2.5	1.57	7.5	[103]
Chiralcel OD	Pindolol	CO_2_/IPA/DEA (60/40/0.1)	40	/	2.5	2.64	16.41	[103]

**Table 13:** Methods and typical results for the enantioseparation of propranolol.

Methods		Mobile Phase	α	Rs	Ref.
CSP	CDMPC-silica hybrid spheres	*n*-Hexane/IPA	1.96	2.75	[61]
	Mono(6^A^-*N*-allylamino-6^A^-deoxy) perphenylcarbamoylated	TEA/MeOH	1.51	4.7	[43]
	Chirobiotic V	MeOH/TEAA	1.12	2.0	[34]
	(+)-(18-crown-6)-2,3,11,12-tetracarboxylic acid based CSP	MeOH /ACN/HOAc/TEA	1.15	2.38	[84]
	Cellulase immobilized via carboxy groups	NaH_2_PO_4_/IPA	4.58	7.04	[88]
CDR	*N*-(4,6-Dichloro-[1,3,5]triazine-2-yl)-l-Val	H_2_O/MeCN/TFA	1.10	9.83	[94]
	(S)-(−)-α,4-dimethylbenzylamine substituted DFDNB	MeCN/TFA/CH_3_CN	1.07	10.07	[92]
	(R)-2-(5-fluoro-2,4-dinitrophenylamino)-3-(methylthio)propanoic acid	CAN/TEA	1.15	14.12	[97]
SFC	Chiralpak AD-*H*	CO_2_/IPA/DEA	1.57	7.5	[103]

## Data Availability

Not applicable.
